# Early morning anopheline mosquito biting, a potential driver of malaria transmission in Busia County, western Kenya

**DOI:** 10.1186/s12936-024-04893-3

**Published:** 2024-03-04

**Authors:** Julius I. Odero, Bernard Abong’o, Vincent Moshi, Sheila Ekodir, Steven A. Harvey, Eric Ochomo, John E. Gimnig, Nicole L. Achee, John P. Grieco, Prisca A. Oria, April Monroe

**Affiliations:** 1 Centre for Global Health Research, Kenya Medical Research Institute, Kisumu, Kenya; 2 Johns Hopkins Bloomberg School of Public Health, Baltimore, MD USA; 3 Division of Parasitic Diseases and Malaria, Centers for Disease Control (CDC) and Prevention, Atlanta, GA USA; 4 Department of Biological Sciences, University of Notre Dame, Eck Institute for Global Health, Notre Dame, IN USA; 5 Johns Hopkins Center for Communication Programs, Baltimore, MD USA

**Keywords:** *Anopheles*, Human behavior, Insecticide Treated Nets (ITNs), Malaria, Night-time observation

## Abstract

**Background:**

Insecticide-treated nets (ITNs) contributed significantly to the decline in malaria since 2000. Their protective efficacy depends not only on access, use, and net integrity, but also location of people within the home environment and mosquito biting profiles. Anopheline mosquito biting and human location data were integrated to identify potential gaps in protection and better understand malaria transmission dynamics in Busia County, western Kenya.

**Methods:**

Direct observation of human activities and human landing catches (HLC) were performed hourly between 1700 to 0700 h. Household members were recorded as home or away; and, if at home, as indoors/outdoors, awake/asleep, and under a net or not. Aggregated data was analysed by weighting hourly anopheline biting activity with human location. Standard indicators of human-vector interaction were calculated using a Microsoft Excel template.

**Results:**

There was no significant difference between indoor and outdoor biting for *Anopheles gambiae *sensu lato (*s.l.*) (RR = 0.82; 95% CI 0.65–1.03); significantly fewer *Anopheles funestus* were captured outdoors than indoors (RR = 0.41; 95% CI 0.25–0.66). Biting peaked before dawn and extended into early morning hours when people began to awake and perform routine activities, between 0400–0700 h for *An. gambiae* and 0300–0700 h for *An. funestus*. The study population away from home peaked at 1700–1800 h (58%), gradually decreased and remained constant at 10% throughout the night, before rising again to 40% by 0600–0700 h. When accounting for resident location, nearly all bites within the peri-domestic space (defined as inside household structures and surrounding outdoor spaces) occurred indoors for unprotected people (98%). Using an ITN while sleeping was estimated to prevent 79% and 82% of bites for *An. gambiae* and *An. funestus,* respectively. For an ITN user, most remaining exposure to bites occurred indoors in the hours before bed and early morning.

**Conclusion:**

While use of an ITN was estimated to prevent most vector bites in this context, results suggest gaps in protection, particularly in the early hours of the morning when biting peaks and many people are awake and active. Assessment of additional human exposure points, including outside of the peri-domestic setting, are needed to guide supplementary interventions for transmission reduction.

## Background

Insecticide-treated nets (ITNs) are one of the few WHO recommended vector control interventions for malaria prevention and models suggest their scale up has contributed substantially to the decline in malaria since 2000 [1]. To sustain the gains achieved in the fight against malaria over the past 20 + years, the global strategy of the World Health Organization (WHO) aims to provide all people at risk with ITNs or indoor residual spraying (IRS) [2, 3]. To achieve and sustain universal coverage, provision of sufficient quantity of nets through available distribution channels is crucial [4]. Whereas access to nets and use are both critical to disrupt human-vector interaction, the former is a primary driver of the latter [5–7], i.e., individuals are more likely to use nets if they have them. Beyond obvious gaps in access and use, the physical integrity of nets and possible human exposure away from the location where nets are intended to be used (i.e., inside houses) provide avenues for human-vector contact and malaria transmission.

Historically, *Anopheles* mosquitoes have been largely nocturnal with feeding by the primary vectors in Africa generally occurring indoors, late at night. In the pre-bed net era, indoor biting by *An. gambiae* and *An. funestus* in Western Kenya were observed to peak three hours and one hour before dawn, respectively [8]. However, changes in mosquito behaviour could be induced by interventions such as ITNs and IRS, reducing their efficacy [9]. Consequently, changes in mosquito biting behaviour, including increased proportions of outdoor biting, early evening biting, and a recent example of daytime biting, have been reported in different settings in sub-Saharan Africa [10–14]. To understand the extent of the changes and develop solutions, an understanding of the biting patterns of mosquitoes and how these overlap with human behaviour and where potential gaps in protection may exist is necessary [15]. Consequently, understanding the distribution of human populations indoors and outdoors, hours in which humans are awake or asleep, and if and when they use ITNs over the course of the night enables a more accurate representation of biting exposure [16]. Such assessment is critical to optimizing existing malaria control interventions and planning new ones [17].

The current study investigated the overlap between mosquito and human activity patterns by location to provide a more accurate measurement of risk. Data described here were collected during the baseline period of a cluster-randomized controlled efficacy trial (cRCT) testing a new vector control product, a spatial repellent and a companion social science study [18]. The study contributes to an improved understanding of sustained malaria transmission in the presence of the current control tools, possible changes in malaria transmission risk with the introduction of new tools and the potential impact and limitations of these tools [15, 19].

## Methods

### Study area

**Fig. 1:**
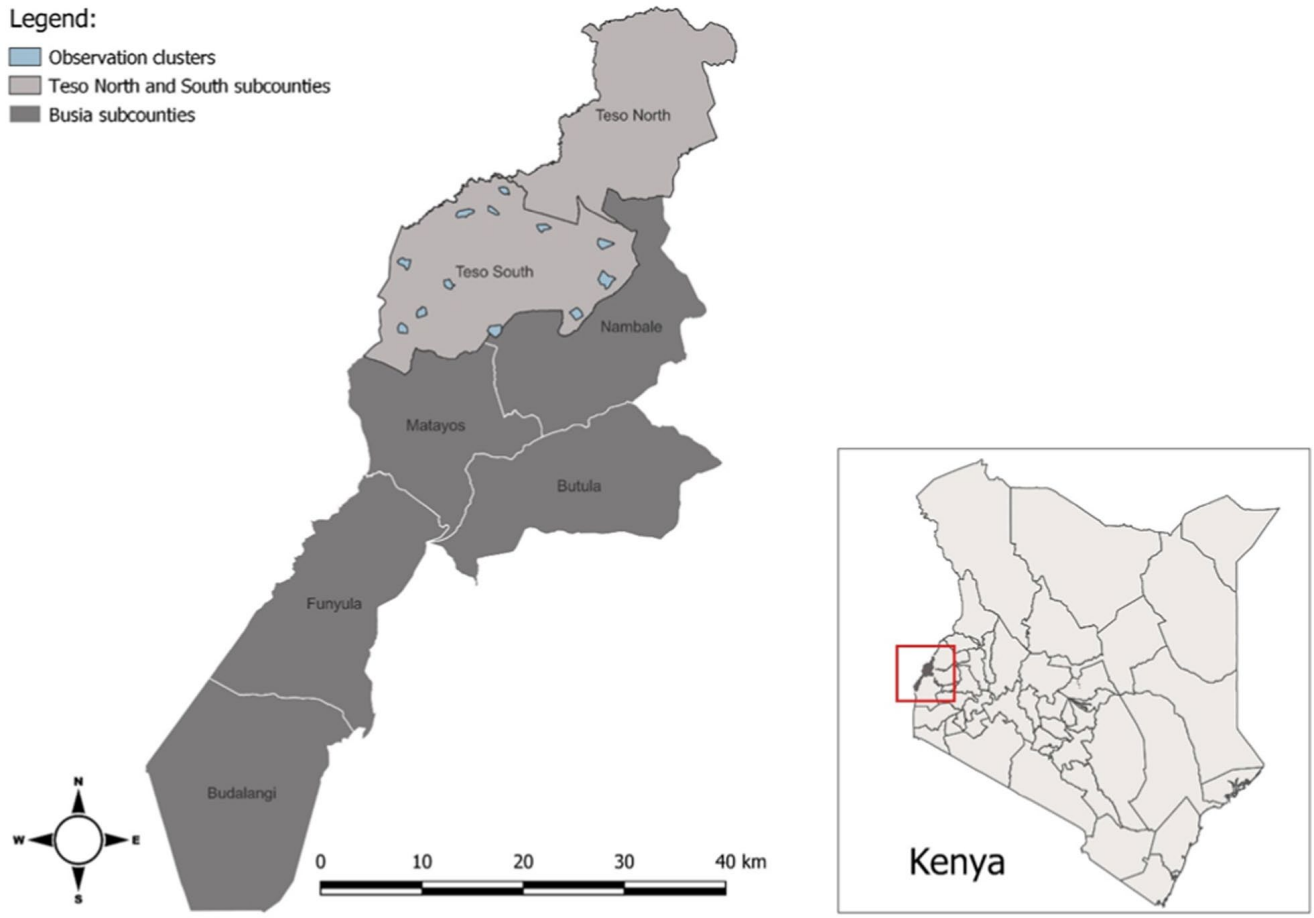
Map of study sites

The study took place in Teso South and part of Teso North sub counties of Busia County, western Kenya (Fig. 1). The study site has been described elsewhere [18]. Briefly, the study population is predominantly of the Iteso ethnic group, lives in scattered homesteads and survives primarily on subsistence farming. Malaria transmission is high and perennial with seasonal peaks during rainy periods in May–June and October–November. The long rains occur from late March to early June and the short rains occur around October through November. The primary mosquito vectors observed in the area include *An. gambiae *sensu stricto (*s.s*.), *Anopheles arabiensis*, and *An. funestus* [20].

### Study design and sampling

The data described in this paper are based on nighttime observation of both human and mosquito activity. Procedures for each are described in detail below. Four compounds were randomly selected from each of 12 villages (48 compounds total) in Teso South and part of Teso North sub counties of Busia County for both entomological and human observation to enable comparability of data on mosquito biting patterns and human activity. Human landing collections (HLC) were conducted in 48 houses and household member observation in 47 of those compounds; one compound was not available for observation. The HLC structures had either mud or cement walls with either grass or iron sheet roofs. All the houses had open eaves and at least two sleepers. A verbal consent to perform mosquito collections was obtained from the household head.

### Anopheline biting behaviour

Anopheline biting behaviour was measured by HLC that occurred indoors and outdoors of a single house within each randomized compound during May and June 2021 before implementation of the spatial repellent intervention. For every HLC collection house, four collectors were recruited either being members of the same household or the neighbouring compounds. They were tested for malaria infection using a malaria rapid diagnostic test seven days before collections began and those testing positive were treated. The collectors were placed on weekly malaria prophylaxis, Mefloquine®, beginning seven days before collections began and continuing up to four weeks after the end of collections. The collectors were trained in mosquito collection techniques and the use of Android tablets for data collection. Mosquito collections were performed between 1700 and 0700 h, covering 14 h total. In each house, collection was conducted for two consecutive nights.

Four collectors worked in two shifts each lasting seven hours, the first shift being from 1700 to 0000 h and the second shift from 0000 to 0700 h. During each shift, collections were performed by two collectors, one of whom sat outdoors, approximately 5 m [21] from the house and the other who sat indoors in the living room. The collectors exposed their legs from the knee down while covering the rest of the body including the arms and the neck. A dimly lit kerosene lamp was placed nearby to provide some light. A torch (flashlight) was used to spot the mosquitoes landing on the exposed legs of the HLC collector. Mosquitoes landing on the exposed limbs were aspirated and transferred into paper cups. Within each collection hour, the collectors worked for 45 min with 15-min breaks between the hours. Collectors rotated their positions between the shifts in the second collection night to minimize the impact of collector bias. During the 15-min breaks in each collection hour, the collectors ran a short questionnaire on an Android tablet to capture information of any mosquito intervention used, presence of open fire, rainfall and if mosquitoes were collected within the last hour. At the end of the questionnaire, the tablet generated a collection code, unique to every collection hour. The collection code, collection date and structure identity were used to label the paper cup for each hour. The collected mosquitoes were provided with a 10% sugar solution soaked in cotton wool to keep alive pending parity dissection in the laboratory. The collected mosquitoes were submitted to the laboratory the following morning for morphological identification and parity dissection.

### Human activity observations

Human activity data were collected by direct observation of residents between 9th and 20th August 2021. Two data collectors, drawn from each compound’s pool of four HLC collectors, were trained as nighttime observers. Before recruiting them as observers, study team supervisors evaluated each data collector’s knowledge about the SR study, the observation method, research ethics, and how to operate the digital tablets and the observation form. The most proficient data collector in each compound was selected to participate in the nighttime observation pre-test and data collection. A pretest was conducted in all 47 participating compounds to identify and address potential challenges. Supervisors reviewed data for quality and provided feedback throughout the data collection process. Prior to the observations, written consent was obtained from each head of compound or their representative. A pre-observation survey documented household membership, ITN ownership, and age, sex, and relationship of each member to the head of compound. At a standardized time, each hour, observers recorded whether each member was at home or away. For those at home, the observer recorded whether that individual was: (1) indoors or outdoors, (2) awake or asleep, and (3) protected or not by an ITN. Also recorded was (4) any activity in which the observed individual was engaged. Data were recorded electronically using tablet-based forms created in CommCare version 2.47.4 (Dimagi, Inc, Cambridge, MA, USA), a health research software program. Built-in skip patterns helped ensure data quality and prevent later entry of any missed observation. The tablets were programmed to upload new data automatically to a secure server every hour.

### Laboratory analysis

All collected *Anopheles* were killed by freezing prior to morphological identification using the keys described by Coetzee [22]. Further species discrimination was performed only for *An. gambiae *sensu lato (*s.l.*) and *An. funestus* groups while all females were analysed for sporozoite infection. Parity dissection was performed on unfed female *Anopheles* to determine parity status according to the standard operating procedures described in the MR4 Methods in *Anopheles* Research [23]. The legs and wings were used in PCR analyses to identify species level members of the *An. gambiae* species complex and *An. funestus* group. The protocol of Scott et al. [24] as described in standard operating procedures in the MR4 Methods in *Anopheles* Research [23] was used for distinguishing between different species of the *An. gambiae s.l.*, while the protocol of Koekemoer et al*.* [25] was used to identify members of the *An. funestus* species group.

### Data analysis

#### Vector behaviour

Analysis of mosquito biting behaviour was performed using R statistical software version 4.2.1. The risk ratio (RR) was used to assess the statistical significance in numbers of mosquitoes biting indoors compared to outdoors. Data were fitted using Generalized Linear Mixed Effects Statistical Models (GLMMs). Since the data were over-dispersed, the package Generalized Linear Mixed Models using Template Model Builder (glmmTMB) was used to fit negative binomial distribution models for the analysis of mosquito numbers. The numbers of female *Anopheles* mosquitoes were assessed as a function of the collection location (indoors or outdoors) as a fixed effect, while collection house was treated as a random effect. Model coefficients were exponentiated to determine the risk ratios (RR) and 95% confidence intervals. Statistical significance level was set at α = 0.05.

#### Human activity observation

Raw observation data were downloaded in CSV format from CommCare into Microsoft Excel 365 where they were cleaned and validated. STATA 13 (StataCorp, College Station, TX, USA) was used for descriptive analysis and to calculate proportions. ITN access was estimated using the approach recommended by the Roll Back Malaria Monitoring and Evaluation Reference Group. This involved first multiplying the number of ITNs in each household by two (one ITN for every two household members). If available ITNs exceeded one for every two household members, all household members were assumed to have access. ITN access was calculated by dividing potential ITN users (those with access to an ITN as defined above) by the total number of study participants [26]. The use to access ratio (UAR) was calculated by dividing the proportion of participants observed to be using an ITN by the proportion with access [15].

#### Human-vector interaction

Human-vector interaction indicators were calculated using the approach described by Monroe et al*.* [15]. Human and mosquito data were entered into an Excel template with inbuilt formulas for measuring and characterizing human vector interaction developed and previously used in Zanzibar [27]. In the formulas used for calculation, ‘π’ is the average proportion of human exposure to vector bites that occurs under certain conditions. ‘I’ denotes Indoors, ‘O’ denotes Outdoors, ‘S’ denotes sleeping space, ‘P’ denotes protected, ‘U’ denotes unprotected, B_I,t_ denotes indoor biting rate at time t, B_O,t_ denotes outdoor biting rates at time t, S_t_ denotes proportion of people in bed sleeping or trying to sleep at time t.

a) Proportion of vector bites occurring indoors for an unprotected individual; (${\pi }_{I,u}$). This indicator represents the maximum possible protection any indoor intervention could provide. It is calculated by summing the weighted indoor vector biting rates (${B}_{I}$) for every hour of mosquito collection by the proportion of people who are indoors (I) at that time and dividing by the sum of indoor and outdoor biting.

$${\pi }_{I,u}=\frac{{\sum }_{t=1}^{24}{B}_{I,t}{I}_{t}}{{\sum }_{t=1}^{24}{B}_{I,t}{I}_{t}+{B}_{O,t{O}_{t}}}$$

b) Percentage of vector bites occurring while asleep indoors for an unprotected individual (${\pi }_{S,u}$). This is an indicator of the maximum possible personal protection an intervention targeting sleeping spaces, such as ITNs, could provide if used as intended. This is calculated by adding the indoor biting rates (${B}_{I}$) every hour for the duration of the mosquito collection period with the estimated proportion of humans sleeping (s) indoors at that time, divided by the summation of indoor and outdoor exposure.

$$\pi _{{s,u}} = \frac{{\sum _{{t = 1}}^{{24}} B_{{I,t}} S_{t} }}{{\sum _{{t = 1}}^{{24}} B_{{I,t}} I_{t} + B_{{O,t}} O_{t} }}$$

c) Percentage of all vector bites directly prevented by using an ITN (${P}_{S}^{*}$). This is calculated as the product of the proportion of exposure occurring while asleep and the personal protection against bites (feeding inhibition) provided by an ITN in use (ρ). ITNs were assumed to prevent 92% of vector bites when in use based on experimental hut trials of PermaNet® 3.0 ITNs in western Kenya.

$$P_{S}^{*} = \rho \pi _{{s,u}} = \frac{{\rho \sum\nolimits_{{t = 1}}^{{24}} {B_{{I,t}} S_{t} } }}{{\sum\nolimits_{{t = 1}}^{{24}} {B_{{I,t}} I_{t} + B_{{O,t}} O_{t} } }}$$

d) Percentage of remaining exposure occurring indoors for a protected ITN user (${\pi }_{I,p})$. This is an indicator of where remaining exposure to vector bites occurs for an ITN user. This is calculated by adjusting the estimate of ${\pi }_{I,u}$ to allow for the indoor personal protection provided by using an ITN.

$$\pi _{{I,p}} = \frac{{\left( {\sum\nolimits_{{t = 1}}^{{24}} {B_{{I,t}} I_{t} } } \right) - \rho \left( {\sum\nolimits_{{t = 1}}^{{24}} {B_{{I,t}} S_{t} } } \right)}}{{\left( {\sum\nolimits_{{t = 1}}^{{24}} {B_{{O,t}} O_{t} + B_{{I,t}} I_{t} } } \right) - \rho \left( {\sum\nolimits_{{t = 1}}^{{24}} {B_{{I,t}} S_{t} } } \right)}}$$

e) Population-wide mean personal protection against biting exposure provided by observed levels of ITN use $\left(C\right)$ in the community $\left({P}_{S,C}^{*}\right)$ calculated as the product of the proportion of the population using an ITN at each hour during the night and the overall personal protection provided by an ITN while it is in use, and accounting for the attenuating effects of exposure when the user is active outside the net.

$$P_{{S,C}}^{*} = \frac{{\rho \sum\nolimits_{{t = 1}}^{{24}} {B_{{I,t}} C_{t} } }}{{\sum\nolimits_{{t = 1}}^{{24}} {B_{{I,t}} I_{t} + B_{{O,t}} O_{t} } }} = \rho \pi _{{S,p}} C$$

## Results

### Malaria vector species diversity and biting patterns

#### Description of mosquito counts by species

A total of 936 female *Anopheles* mosquitoes were collected indoors and outdoors of 48 houses over a two-day collection period per house. Of these, 727 (77.7%) were *An. gambiae s.l*., 186 (19.9%) *An. funestus* Group, 21 (2.2%) *An. coustani* and 2 (0.2) *Anopheles rufipes*. Ninety-three percent (N = 635) of the *An*. *gambiae s.l.* were confirmed to be *An. gambiae s.s.* by polymerase chain reaction (PCR) with the remaining (7%) being *An. arabiensis*. All the *An. funestus* (N = 107), tested by PCR were confirmed to be *An. funestus s.s.* Details of the collected mosquitoes by abdominal status, distribution indoors and outdoors and parity status are provided in Table 1. There was no significant difference in the proportion of parous *An. funestus* compared to *An. gambiae* (χ^2^ = 0.15, df = 1, p = 0.70). Additionally, no significant difference was observed in the distribution of parous and nulliparous mosquitoes by trapping location, either indoors or outdoors (χ^*2*^ = 0.06, df = 1, p = 0.81) (Table 1).

**Table 1:** Numbers and parity status of anopheline mosquitoes collected indoors and outdoors

*Anopheles* species	Trapping location	Abdominal status					Parity		
		Fed	Gravid	H. Gravid	Unfed	Total N (%)	Parous	Nulliparous	Parity Rate
*An. gambiae*	Indoors	134	16	11	221	382 (52.5)	150	58	71.5%
	Outdoors	106	7	17	215	345 (47.5)	143	59	
*A. funestus*	Indoors	39	2	15	75	131 (70.4)	55	21	73.9%
	Outdoors	15	3	4	33	55 (29.6)	27	8	
*An. coustani*	Indoors	3	0	1	5	9 (42.9)	3	1	55.6%
	Outdoors	2	0	2	8	12 (57.1)	2	3	
*An. rufipes*	Indoors	1	0	0	1	2 (100.0)	2	0	100%
	Outdoors	0	0	0	0	0 (0.0)	0	0	
Total N (%)		300 (32.1)	28 (3.0)	50 (5.3)	558 (59.6)	936	382 (71.8)	150 (28.2)	532

#### Indoor and outdoor biting rates

The mean biting rate of *An. funestus* per person per night was 1.36 indoors and 0.57 outdoors respectively. The mean biting rates of *An. gambiae* was measured at 3.98 indoors and 3.59 outdoors, while that of *An. coustani* was 0.09 indoors and 0.13 outdoors. Significantly lower numbers of *An. funestus* were observed to bite outdoors compared to indoors (RR = 0.41, 95%CI: 0.25–0.66, P = 0.0002). No significant difference was observed in the numbers of *An. gambiae* complex (predominantly *An. gambiae s.s.*) and *An. coustani* biting indoors and outdoors (RR = 0.82, 95%CI: 0.65–1.03, p = 0.09 and 1.86, 95%CI: 0.54–6.40, p = 0.33, respectively) (Table 2).

**Table 2:** Comparison of *An. funestus*, *An. gambiae* and *An. coustani* biting rates indoor and outdoor

*Anopheles* species	Sampling location	Mean (95%CI) bites per person per night	RR (95%CI)	*P* values
*An. funestus*	Outdoor	0.04 (0.03–0.06)	0.41 (0.25–0.66)	< 0.01
	Indoor	0.10 (0.07–0.13)	Ref.	
*An. gambiae* Complex	Outdoor	0.27 (0.22–0.32)	0.82 (0.65–1.03)	0.09
	Indoor	0.30 (0.24–0.35)	Ref.	
*An. coustani*	Outdoor	0.01 (0.00–0.01)	1.86 (0.54–6.40)	0.33
	Indoor	0.01 (0.00–0.01)	Ref.	

### Nighttime human location and sleeping patterns

A total of 328 people were observed across the 47 compounds. Participants were approximately evenly split (p = 0.180) by sex across the various age groups as shown in Table 3.

**Table 3:** Demographic characteristics of household members

Household members	Male	Female	Total
	153 (46.65%)	175 (53.4%)	328
< 1 year	5 (0.6%)	1 (0.8%)	6 (1.8%)
1–4 years	25 (16.3%)	16 (9.1%)	41 (12.5%)
5–9 years	15 (9.8%)	18 (10.3%)	33 (10.1%)
10–17 years	38 (24.8%)	47 (26.9%)	85 (25.9%)
18–59 years	60 (39.2%)	81 (46.3%)	141 (43.0%)
≥ 60 years	10 (6.5%)	12 (6.9%)	22 (6.7%)

#### Time spent away from home

**Fig. 2:**
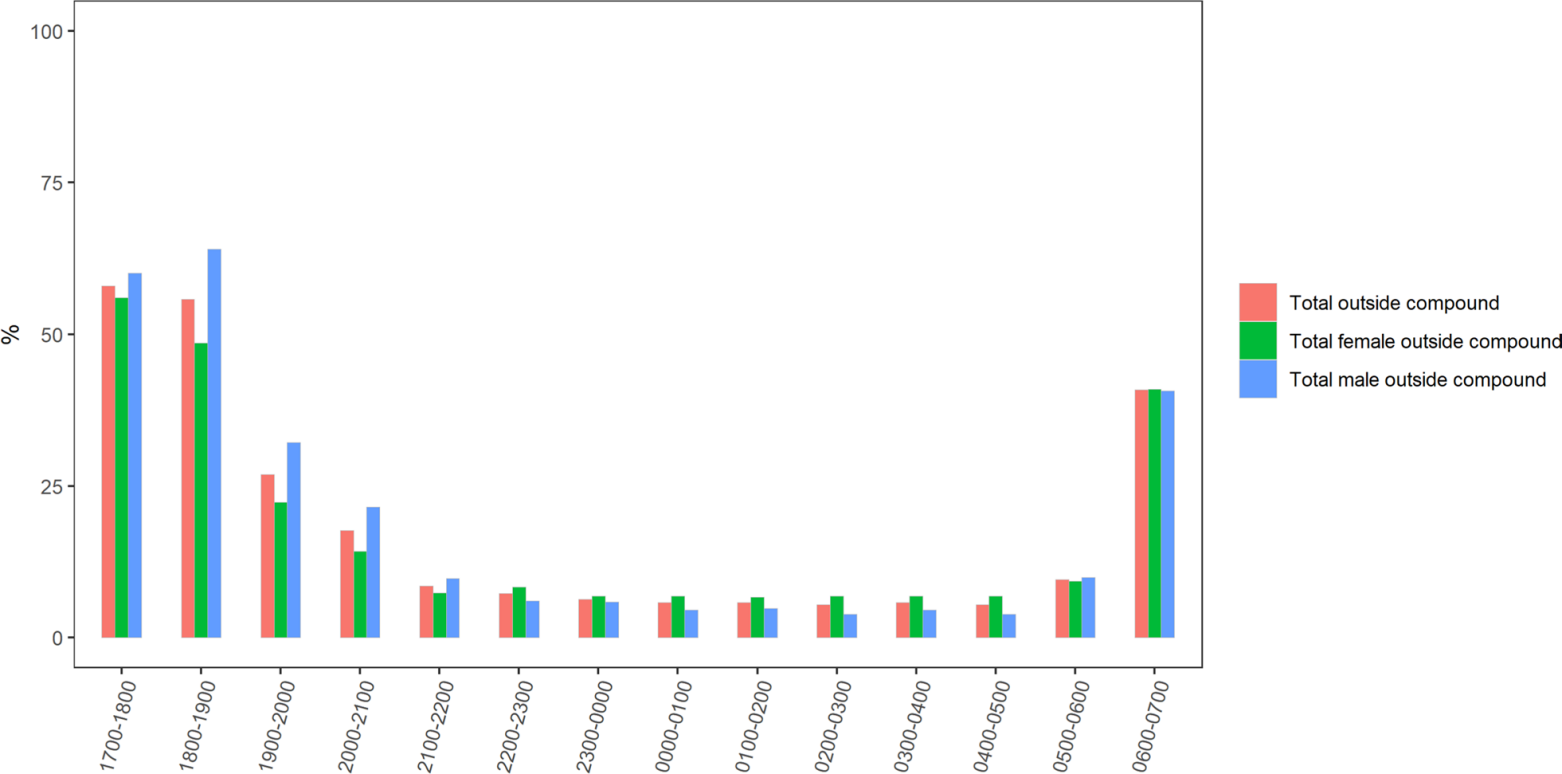
Percentage of study population away from home, by hour

The percentage of the study population observed as away from home peaked in the early evening and slowly declined from 58% at 1700 h to 25% at 2000 h (Fig. 2). The percentage dropped to 10% by 2200 h, remained at that level until 0500 h, then rose again to 40% between 0500 and 0700 h. Between 1700 and 2200 h, the percentage of males away from home was higher than that of females with a peak of over 60% of males away in the early evening, between 1700 and 1900 h. From 2200 to 0700 h, patterns were similar for males and females.

#### Time spent in the peri-domestic space

Among participants recorded as being at home, approximately 75% (246/328) of observed participants were outdoors between 1700 and 1900 h, declining to approximately 25% between 1900 and 2000 h. The percentage indoors rose correspondingly, with indoor population reaching and remaining at 90% (295/328) from 2000 to 0400 h (Fig. 5).

#### ITN access and use

**Fig. 3:**
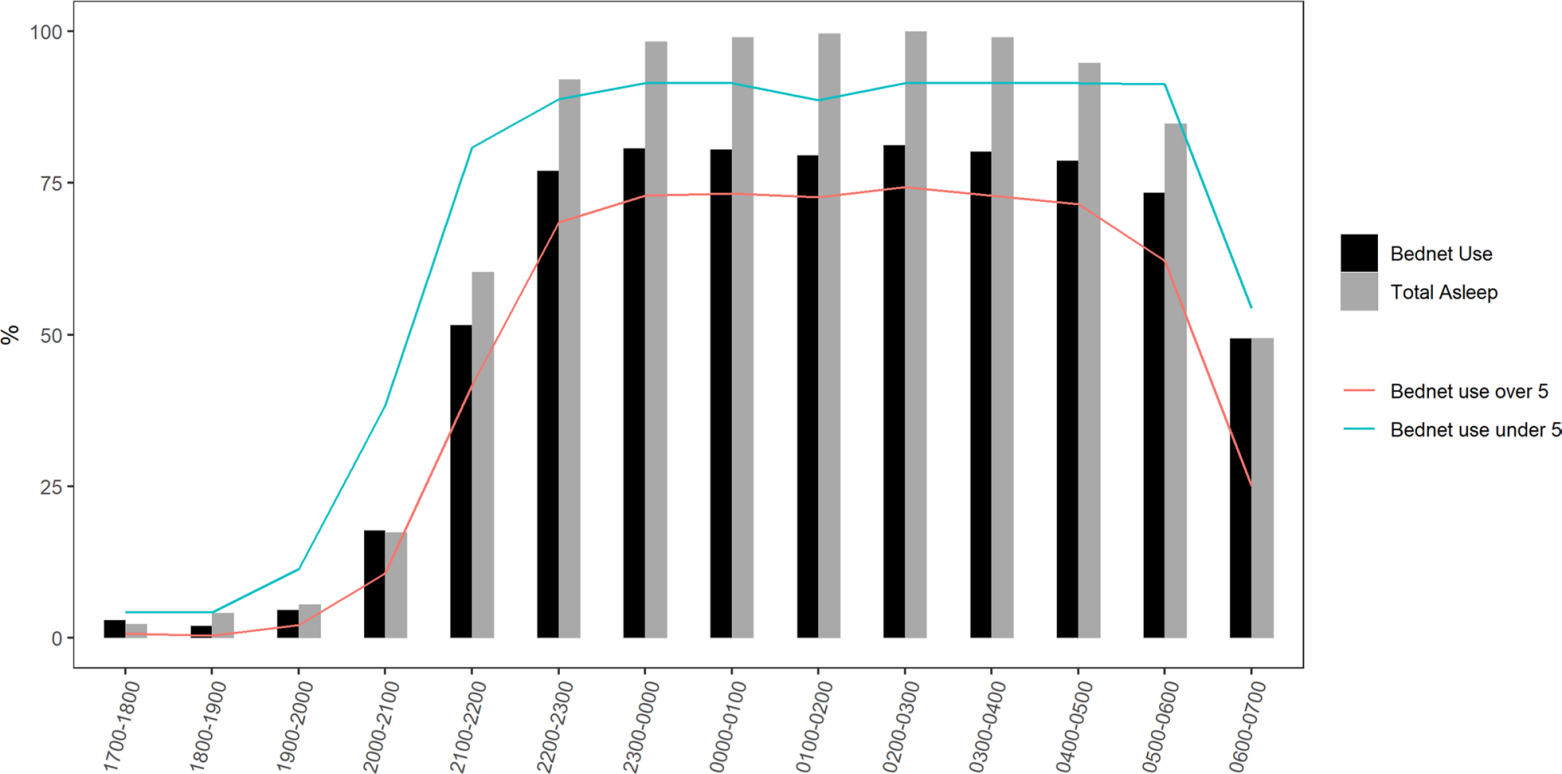
Percentage of study population protected by an ITN by hour and age (< 5 and ≥ 5yrs)

Based on the WHO recommendations of one net for every two household members, 98% of those observed (323/328) had access to an ITN. Among those with access to an ITN, approximately 90% (293/323) used one at some point during the night. Usage was lowest (9.8% 29/293) between 1700 and 2000 h, increasing to 20% (58/293) by 2100 h and 80% (234/293) by 2300 h, and remaining at that level until 0500 h when people began to wake up. Over 90% of children under 5 slept under a net compared to 70% of older children and adults (Fig. 3).

### Frequency of common activities by time

**Fig. 4:**
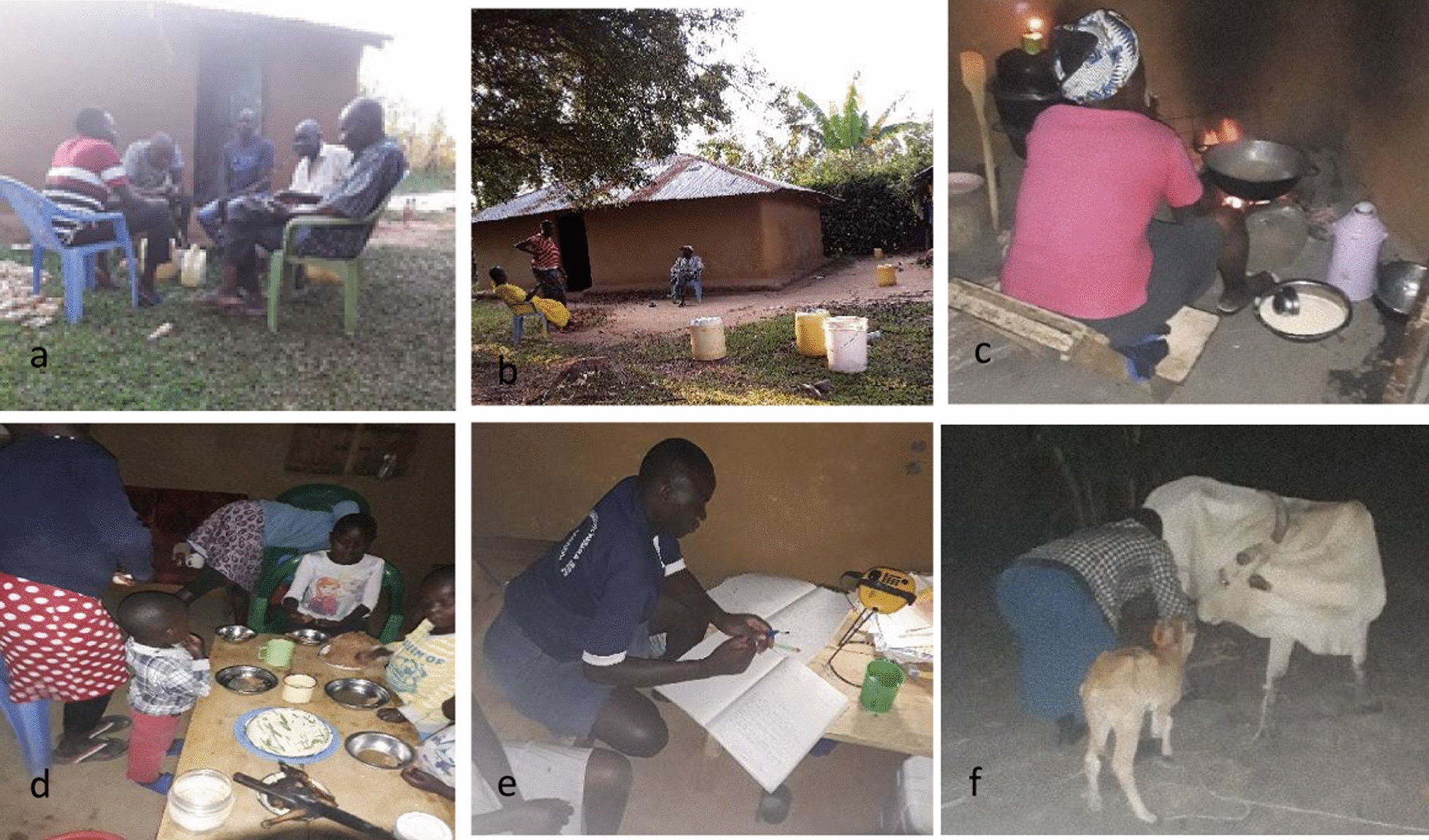
Evening (**a**, **b**), night (**c**, **d**) and morning (**e**, **f**) activities carried out by the study population. Photo a, a group of men drinking local brew, photo b, women chatting/socializing, photo c, a woman cooking, photo d, children eating, photo e, a student studying and photo f, a woman milking a cow

Socializing, eating, cooking, reading, playing, and finishing other household chores were the main activities observed outdoors and indoors in the population during evening hours between 1700 and 2200 h (Fig. 4). Other common activities included watching TV and listening to the radio. During late night hours, very few activities were recorded as most people were sleeping. Activities peaked again in the early morning when people began morning routines. Women and girls spent time cooking, socializing, eating, and completing household chores. Men and boys engaged in playing, eating, reading, socializing, watching television, and listening to radio. Of all activities recorded during this time, 68.2% (n = 690) occurred indoors compared to 31.8% (n = 321) outdoors.

#### Evening activities (1700–2200 h)

The main activities recorded indoors in the evening between 1700 and 2200 h, were eating (98/690), reading (79/690), cooking (76/690), socializing (71/690), and watching television (54/690). Other activities were participants relaxing (40/690), listening to radio (33/690), finishing household chores (31/690) and playing (30/690). The same activities were also recorded to have occurred outdoors with the main activities outdoors being playing (51/321), socializing (38/321), finishing household chores (36/321), eating (13/321), cooking (9/321), and listening to radio (8/321) among others. (Fig. 4a, b).

#### Night activities (2200–0400 h)

At night between 2200 and 0400 h, minimal activities were recorded as most participants were sleeping. Of the activities occurring at this time, most of them occurred indoors which included reading (7/690), listening to radio (6/690), relaxing (5/690) socializing (4/690) and watching television (3/690). (Fig. 4c, d).

#### Morning activities (0400–0700 h)

In the morning between 0400 and 0700 h, resumption of routine activities was observed. The main activities that were recorded indoors were cooking (10/690), preparation for school (6/690), reading (5/690) listening to radio (4/690). Other activities occurring indoors were praying, resting, relaxing, and watching television. Outdoor activities at this time were mainly preparing for school (6/321), cleaning the compound, digging, and milking cows. (Fig. 4e, f).

### Patterns of human-vector interactions

**Fig. 5:**
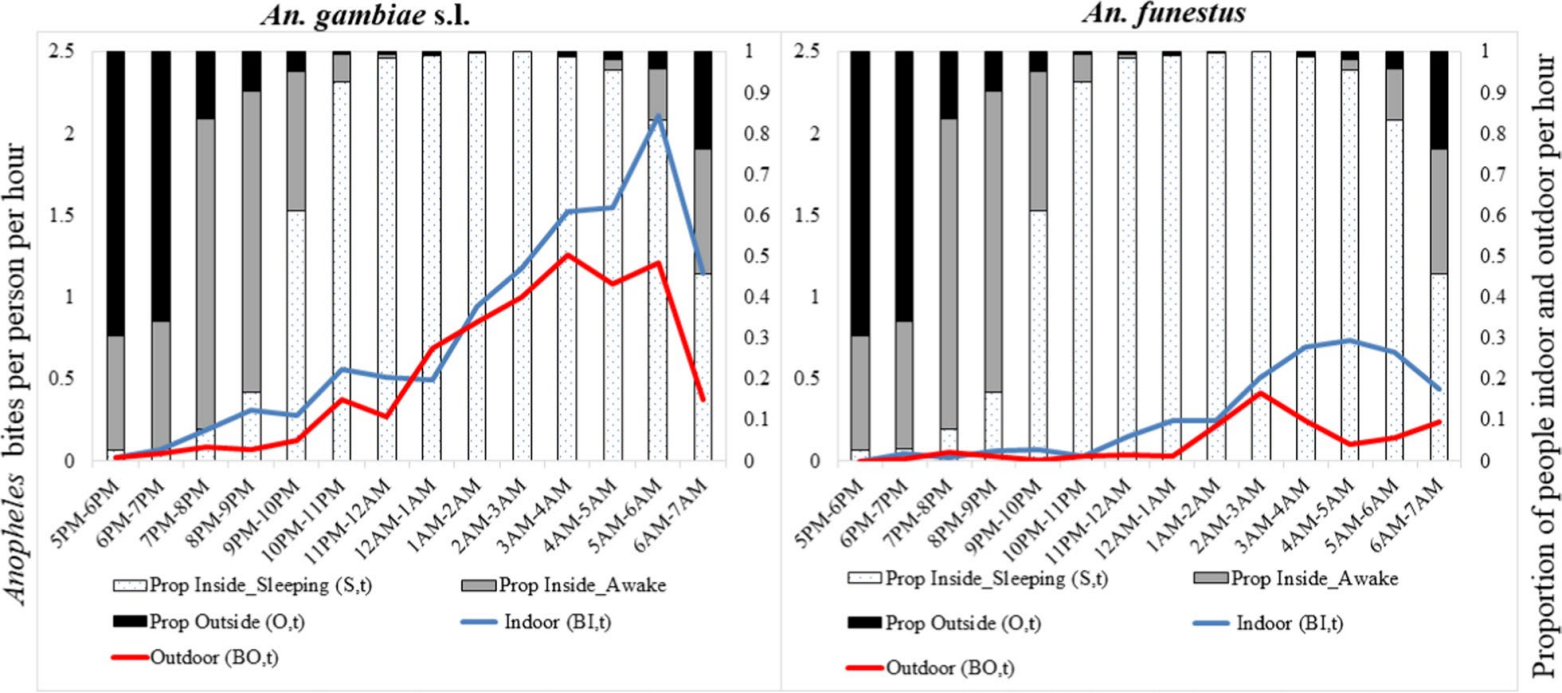
Human location overlaid with raw (directly measured by HLC) indoor and outdoor mosquito biting rates

The bulk of biting by *An. gambiae* indoors occurred at a time when most people were asleep with the peak biting at dawn. Additional exposures occurred indoors at dusk before bedtime. Negligible exposure to bites by *An. gambiae* was observed to occur outdoors at dusk but with increased frequency at dawn. Exposure to bites by *An. funestus* was nearly zero for the first half of the night but began to increase at around midnight with the peak biting occurring at dawn. The highest bite rates by *An. funestus* were experienced indoors at a time when most people were indoors and asleep. Additional exposure was observed towards morning for people who were awake indoors and outdoors. Biting by both *An. gambiae* and *An. funestus* indoors and outdoors was still high in the morning at the time when collection ceased, and almost half of the people being observed were already awake (Fig. 5).

**Fig. 6:**
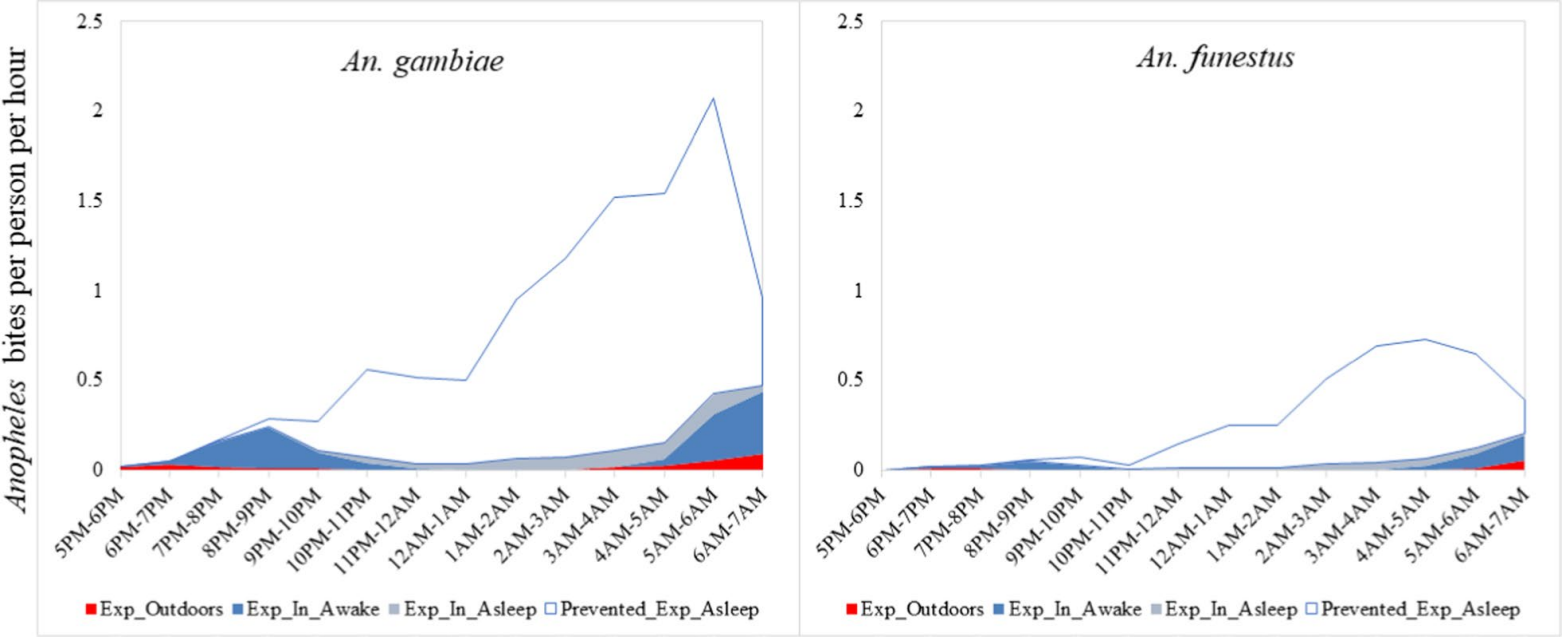
Human exposure to malaria vectors prevented by use of an ITN (white area) and exposure that remains indoors asleep (grey area), indoors awake (blue area) and outdoors (red area)

The proportions of directly measured *An. gambiae* and *An. funestus* biting were 0.59 and 0.71 indoors and 0.41 and 0.29 outdoors, respectively (Table 4). When accounting for human location, for an unprotected individual in the peri-domestic space, the proportion (${\pi }_{I,u}$) of bites that occurred indoors was 0.98 for both *An. gambiae* and *An. funestus*. Based on HLC, the proportion (${\pi }_{S,u}$) of bites occurring during the hours when unprotected people would have been asleep was 0.86 for *An. gambiae* and 0.89 for *An. funestus*. While adjusting for human behaviour and assuming a protective efficacy of 92.0% for ITNs, the proportion (${P}_{S}^{*}$) of all bites prevented by using an ITN was estimated at 0.79 for *An. gambiae* and 0.82 for *An. funestus*. Of the remaining bites for a protected ITN user (${\pi }_{I,p}$), 0.88 and 0.87 were estimated to occur indoors for *An. gambiae* and *An. funestus,* respectively. The remaining exposures for a protected ITN user were distributed as follows, proportion (${\pi }_{S,P}$) of exposure occurring while asleep for a protected net user of 0.34 and 0.39 for *An. gambiae* and *An. funestus,* respectively and exposure occurring indoors while not asleep measured at 0.55 and 0.49 for *An. gambiae* and *An. funestus,* respectively. The proportion (${P}_{S,C }^{*}$) of exposure prevented by current levels of ITN use in the population was estimated at 60% and 62% for *An. gambiae* and *An. funestus,* respectively ((Fig. 6).

**Table 4:** Human-vector indicators

Category	Indicator	*An. gambiae*^*1*^	*An. funestus*
Directly Measured Biting	Proportion biting indoors	0.59	0.71
	Proportion biting outdoors	0.41	0.29
Behaviour-Adjusted Exposure—Unprotected Individual	Proportion of vector bites occurring indoors for an unprotected individual (${\pi }_{I,u}$):	0.98	0.98
	Proportion of vector bites occurring while asleep for an unprotected individual (${\pi }_{S,u}$):	0.86	0.89
Behaviour- Adjusted Exposure—ITN-User	Proportion of all vector bites prevented by using an ITN (P*):	0.79	0.82
	Proportion of human exposure occurring while asleep for a protected user of an ITN (${\pi }_{S,P}$):	0.34	0.39
	Proportion of human exposure occurring indoors but not asleep	0.55	0.49
	Proportion of remaining exposure occurring indoors for a protected user of an ITN (${\pi }_{I,p}$):	0.88	0.87
Behaviour-Adjusted Exposure—Population Mean	Proportion of exposure prevented by current levels of ITN use in the population (P*S,C):	0.60	0.62

^1^Anophelines captured using HLC from 48 houses during 1700—0700 h from May 31st to June 18th, 2021

## Discussion

Characterizing the timing of interactions between humans and anopheline mosquitoes in the context of existing malaria vector control interventions is important for estimating the benefit level and gaps in protection [26, 28]. It further informs the development and targeting of complementary interventions that meet people’s needs and preferences [27]. The timing of human activities, and sleeping behaviours in particular, has a strong modulating effect upon human-mosquito contact and the effectiveness of ITNs [16]. This work adds to a growing trend towards integrating human and vector behaviour data to get a more complete picture of risk [19, 29–33]. By bringing together information on net access and use, human location and sleeping patterns, and indoor and outdoor vector biting behaviour, it was possible to identify times and locations where people are likely to need additional protection. This is particularly important in western Kenya where high levels of malaria transmission persist, despite good coverage with core vector control interventions.

In endemic regions with high late-night indoor *Anopheles* biting rates, ITNs are an effective tool for malaria prevention [1]. Consistent with previous studies in western Kenya, biting by *Anopheles* mosquitoes in the study area was observed to occur mostly indoors, late at night [30] thus protecting ITN users from most biting. Similar observations have been made in other endemic settings [29, 34]. Other research findings have however reported increased outdoor biting malaria vectors due to increased use of vector control interventions indoors [14, 35–37]. The findings suggest that in western Kenya a high proportion of anopheline biting continues to occur indoors and late at night when most people—like those observed in the study area—would be sleeping. As a result, ITNs should continue to offer effective protection as long as they are used as intended and the insecticides in use remain effective. However, late-night indoor biting was observed to culminate in early morning biting, which corresponds to a time when many people are no longer under the protection of ITNs.

Thus, despite high ITN access and use, gaps in protection exist even for regular ITN users. This study demonstrated that risk remains [26], due in large part to the proportion of *An. funestus* and *An. gambiae* biting that occurs during morning hours when the study participants were observed to be indoors, awake, and had exited their ITNs to engage in routine morning activities. These gaps/risks are beyond the protective efficacy of any ITN, including new generation nets, since they are based on possible changes in mosquitoes biting. Consistent with these results, previous studies in western Kenya that extended HLC collections until 1100 h reported continued biting activity by *An. funestus* into late morning hours [38, 39]. Elsewhere, morning exposure to anopheline bites have been previously reported in Benin and Burkina Faso [16, 40]. Given reports of day-biting *An. funestus* in other regions [11, 41], additional studies are recommended to assess the daytime risk to malaria vectors.

Further, a large percentage of participants were recorded to be away from home, during the early evening and morning hours. In the early morning hours between 0500 and 0700 h, a time of concern for indoor and outdoor vector biting, the percentage of people away from home rose to 40%. Indicators of human-vector interaction were calculated for the peri-domestic space, inside and directly outside of homes, however time away from home represents another potential gap in protection. While this study did not document human activities away from home, studies in Ghana, Uganda, and Zanzibar have identified routine social and economic activities as well as all-night funerals weddings, religious ceremonies, and other large community gatherings, as common reasons for being away from home [42–44]. Similar activities are common in western Kenya and potentially offer additional risks for malaria transmission. Characterizing and addressing the risk of transmission associated with such social gatherings and other occupational engagements away from home will be critical for targeted malaria control.

To interrupt malaria transmission within the study population, a variety of interventions adaptable to changing vector behaviour and human activity profiles are needed in the malaria control toolbox. Novel malaria control interventions such as spatial repellent (SR) products and attractive toxic sugar baits (ATSBs) which are presently under evaluation for efficacy [18, 45] could be valuable additions to ITNs in sustaining the gains in the fight against malaria. For example, spatial repellents protect people in the surrounding space through continual release of a volatile active ingredient [46]. Spatial repellents have the potential to provide protection any time of day or night in the locations where they are in use, and if proven efficacious, could help to fill the gap identified in the early morning when people are no longer under the protection of a net.

## Study limitations

Like many other evaluations, this study was not devoid of limitations. The assessment of human-vector interaction pairs mosquito and human activity data, which should be collected at or near the same time. However, due to operational challenges during the COVID-19 pandemic, the two methods were conducted at different times with the national mass net campaign happening in between. The observed high levels of access and use of ITNs may be attributable to the mass net distribution campaign that happened shortly before household observations began. Also, the data presented is from a cross-sectional survey that does not account for seasonality in mosquito or human behaviour. Human-vector indicators provide useful indications of exposure patterns but do not capture all nuances related to potential gaps in protection and require certain assumptions. For example, estimates of personal protection provided by an ITN while in use came from experimental hut trials data and may not reflect personal protection provided in real-world conditions over time.

## Conclusions

This study integrated data on malaria mosquito biting and nighttime human behaviour to better understand potential exposure patterns in western Kenya. Gaps in ITN protection can occur when human activities are incompatible with ITN use or when mosquitoes adapt their biting patterns to hours and contexts inappropriate to such use. In this study, most exposure to malaria mosquito bites was found to occur indoors during times when ITNs can provide protection, underscoring the continued benefit of effective ITNs in this context. However, it also identified an important gap in protection—peak biting occurred in the early morning when many people were already awake and no longer protected by an ITN. Additional research is needed to better understand the extent to which biting may continue into the morning beyond 07:00 h when collections ended. Further, while HLC was carried inside and directly outside of homes, a large proportion of the human population was observed to be away in the evening and early morning hours. It will be important to further characterize exposure patterns away from home and to identify appropriate complementary prevention measures. Several promising vector control interventions are currently under evaluation that could help to address these gaps. This study adds to a growing body of evidence on the importance of integrating entomological and human behavioural data to understand context-specific gaps in protection.

## Data Availability

The datasets used and/or analysed during the current study are available from the corresponding author on reasonable request.

## Abbreviations

KEMRI: Kenya Medical Research Institute
CDC: Centers for Disease Prevention and Control
ITN: Insecticide Treated Nets
HLC: Human landing collection
IRS: Indoor residual spraying
SR: Spatial repellant
ELISA: Enzyme-linked immunosorbent assay
PCR: Polymerase chain reaction
UAR: Use to access ratio
SERU: Scientific Ethics Review Unit
ERC: Ethical Review Committee
